# Long-term effects of face-to-face supervision versus telephone supervision during a community-based pulmonary rehabilitation programme in people with COPD using minimal equipment: a randomized controlled trial

**DOI:** 10.1186/s12875-025-02962-3

**Published:** 2025-08-27

**Authors:** Cristina Sacristán-Galisteo, Tamara del Corral, Carmen Gómez-Pesquera, Ricardo Rodríguez-Barrientos, María Fontana-Campos, Ane Arbillaga-Etxarri, Ibai López-de-Uralde-Villanueva, José Miguel Rodríguez-Gónzález-Moro, Patricia Martín-Casas

**Affiliations:** 1 Doctoral Program in Healthcare, Faculty of Nursing, Physiotherapy and Podiatry, Complutense University of Madrid, Madrid, Spain; 2 Las Fronteras Healthcare Center, Primary Care Assistance Management, Madrid, Spain; 3 Department of Radiology, Rehabilitation and Physiotherapy, Faculty of Nursing, Physiotherapy and Podiatry, Complutense University of Madrid, Instituto de Investigación Sanitaria del Hospital Clínico San Carlos (IdISSC), Madrid, Spain; 4 Unidad de Investigación, Gerencia de Atención Primaria, SERMAS, Madrid, Spain; 5 Department of Preventive Medicine, Hospital Universitario Infanta Sofía, Madrid, Spain; 6 Deusto Physical TherapIker, Physical Therapy Department, Faculty of Health Sciences, University of Deusto, Donostia-San Sebastian, Spain; 7 Department of Pneumology, Hospital Universitario Príncipe de Asturias, Alcalá de Henares, Madrid, Spain

**Keywords:** Chronic obstructive pulmonary disease, Pulmonary rehabilitation, Functional exercise capacity, Long-term effects, Exercise training

## Abstract

**Background:**

Pulmonary rehabilitation is a key and effective treatment for people with chronic obstructive pulmonary disease (COPD) although lack of accessibility is a barrier. The aim of this study was to compare the effects of face-to-face supervision (FFS) with those of telephone supervision (TS) during a community-based pulmonary rehabilitation programme to increase functional exercise capacity in people with COPD using minimal equipment. In addition, physical activity level, health-related quality of life, psychological status, exercise self-efficacy, number of exacerbations and adherence were analysed.

**Methods:**

A single-blind randomized clinical trial was designed, allocating 80 patients from primary care setting 1:1 to FFS (*n* = 40) or TS (*n* = 40) rehabilitation programme. The intervention lasted 13 weeks and was composed of a structured education and exercise training. The variables evaluated were functional exercise capacity (6-minute walk distance [6MWD]), physical activity level (steps/day), health-related quality of life (COPD Assessment Test [CAT]), psychological status (Hospital Anxiety and Depression Scale), exercise self-efficacy (Exercise Self-Efficacy Scale), number of exacerbations and adherence (Adherence to Treatment of Physiotherapy Scale).

**Results:**

Eighty participants were included in this study. Compared with the TS group, the FFS group presented improvements in the 6MWD of 28.8 m (95%CI 12.3–45.3, *p* < 0.01) after the intervention, 19.15 m (95%CI 2.7–35.6, *p* < 0.05) at 6 months, and 28 m (95%CI 11.5–44.5, *p* < 0.01) at 12 months, as well as the CAT score (mean: -4.2, -3.1, and − 4.6 points) respectively. Compared with the TS group, the FFS group performed an increased number of steps/day at the end of the intervention and at 12 months and had a reduced anxiety level at the 6-month follow-up. No differences in depression level, exercise self-efficacy, number of exacerbations or adherence were observed.

**Conclusions:**

Compared with TS, FFS was superior for improving functional exercise capacity, health-related quality of life and physical activity levels in the short and long term in individuals with COPD.

**Trial registration:**

ClinicalTrials.gov ID: NCT05565872 (https://clinicaltrials.gov/study/NCT05565872). Initial release: 09/27/2022.

**Supplementary Information:**

The online version contains supplementary material available at 10.1186/s12875-025-02962-3.

## Background

Chronic obstructive pulmonary disease (COPD) is one of the main non-communicable diseases worldwide. The clinical presentation is the presence of progressive dyspnoea, chronic cough accompanied or not by expectoration, fatigue and frequent exacerbations [1]. These symptoms are associated with a decrease in functional exercise capacity, lower levels of physical activity, high levels of anxiety and depression, and a deterioration in health-related quality of life (HRQoL) [1–4]. Low levels of physical activity and limitation of functional exercise capacity are a prognostic indicator and suffer progressive deterioration due to vicious cycle of physical inactivity, deconditioning and dyspnoea [1, 4, 5]. Thus, designing interventions to improve the functional exercise capacity and to adopt a more active lifestyle is a major goal in COPD management.

To this end, pulmonary rehabilitation (PR) programmes are recommended in clinical practice guidelines for COPD patients and has been shown to be one of the most effective treatments for people with COPD [1, 6, 7]. The recommended standard for PR programmes is the centre-based supervised programmes conducted in a hospital centre using specialized equipment [8, 9]. However, < 5% of individuals with COPD having access to it [10]. This inaccessibility is driven not only by systemic barriers—such as inadequate infrastructure, limited availability of trained healthcare professionals, long waiting lists, and insufficient public funding—but also by social and individual factors. These include limited patient awareness of the benefits of rehabilitation, lack of support from family or caregivers, financial hardship, occupational demands, and the presence of comorbidities such as depression or low motivation [11, 12].

To overcome these barriers, alternative models have been developed, such as home or community-based telerehabilitation using minimal equipment for training, which can be complemented by various e-health tools (mobile applications or telephone calls for monitoring and supervision) [9, 13]. These alternative PR programmes can be characterised by exercise training involving outdoor walking and exercises with elastic bands or bodyweight exercises, all of which require minimal equipment [9, 13, 14]. Preliminary evidence shows that these programmes can produce improvements in exercise capacity, and HRQoL, compared with equipment-based programmes, and may be an alternative to center-based PR [9, 13, 14]. The main benefits of these programmes include improved accessibility, particularly in rural or remote areas with limited access to hospital-based services, and greater cost-effectiveness, as they do not require specialised equipment for their implementation [15, 16]. However, their limitations include the lack of consensus regarding the essential components of these programmes and the need for further clinical studies to support their long-term effectiveness [13].

In this regard, the Urban Training™ methodology stands out. It consists of designing outdoor walking circuits with varying intensity levels, located within the patient’s community environment, to promote physical training [17]. This approach combines an initial motivational interview with tailored exercise recommendations to guide progression through the circuits over several weeks, along with telephone follow-up consisting of one to four calls, depending on the patient’s level of motivation [18]. This model has been shown to improve long-term physical activity levels with minimal telephone supervision (TS) during exercise training [18]. However, no effects were observed on functional exercise capacity, HRQoL, exacerbations, or psychological status; this may be due to insufficient supervision [18]. Given these limitations, this study designed an intervention that combined physical training through community-based urban circuits with structured educational sessions and enhanced supervision. It was hypothesized that adding face-to-face supervision (FFS) would lead to improved health outcomes compared to TS.

Therefore, the main objective of this study was to evaluate the effects of FFS versus TS during a community-based PR programme using minimal equipment on functional exercise capacity in the short, medium and long term in individuals with COPD. The secondary objectives were to analyse the effects on physical activity levels, HRQoL, psychological status, exercise self-efficacy, number of exacerbations and adherence.

## Methods

### Study design

This is a prospective, multicentre, single-blind randomized clinical trial, with two parallel groups (FFS group vs. TS group) registered at clinicalTrials.gov ID: NCT05565872 (Initial release: 09/27/2022) and reported according CONSORT statement and its extension for non-pharmacological interventions. The trial protocol and statistical analysis plan are accessible at the following website https://clinicaltrials.gov/study/NCT05565872. Participants were allocated 1:1 to the FFS group or TS group using random block sizes.

Randomization was performed via a table of random sequences generated with GraphPad©. Randomization was carried out by a researcher who was not involved in the other procedures. The physiotherapist in charge of carrying out the intervention assigned the participants to the corresponding group via a secure computer file containing the randomization sequence. All evaluations were performed by the same investigator who was blinded to the randomization.

**Fig. 1:**
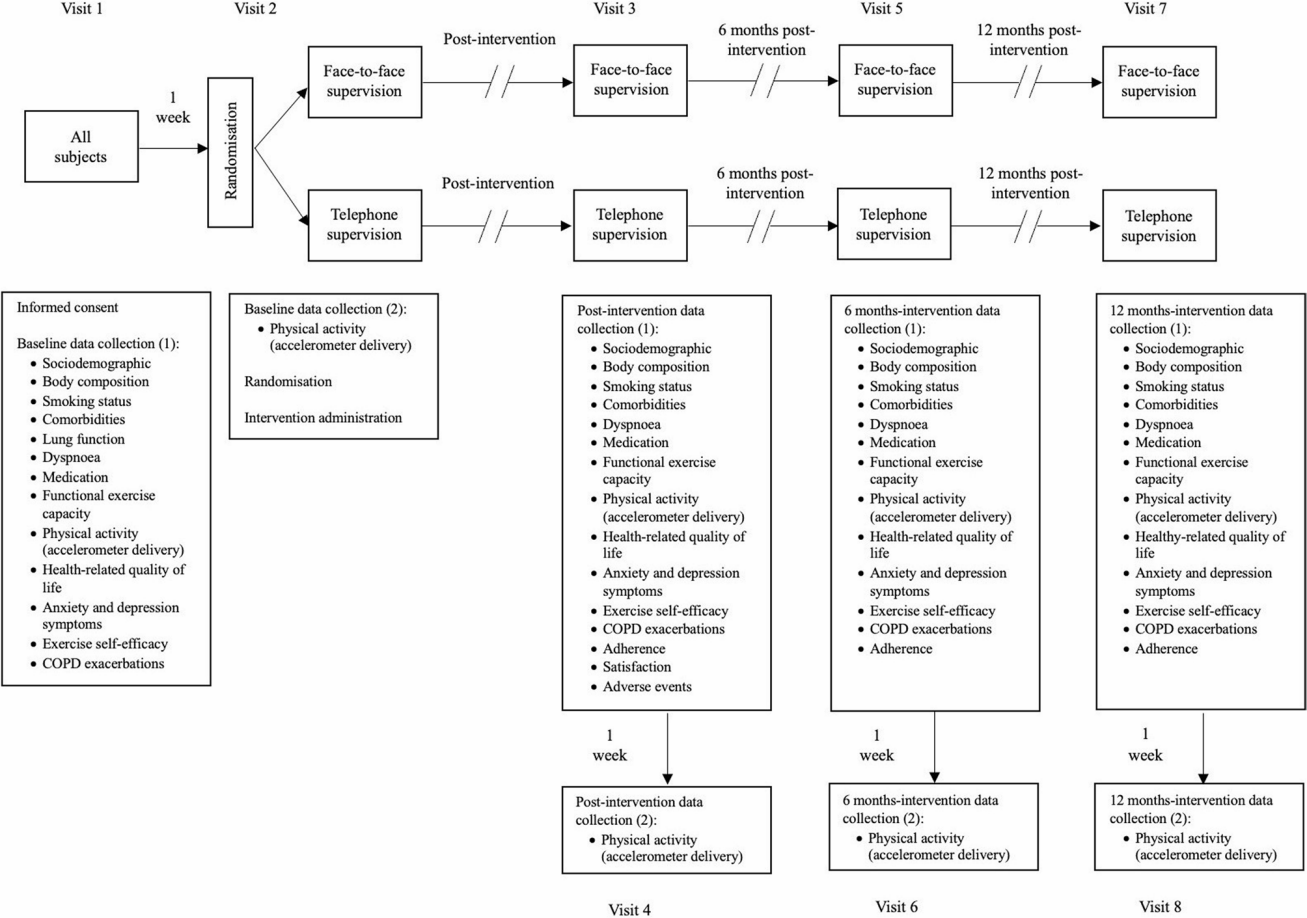
Timeline of study visits and assessments. Abbreviations: COPD, chronic obstructive pulmonary disease

The study consisted in eight visits: enrolment and baseline data collection; randomization and intervention administration, postintervention data collection and 6 and 12-months data collection (Fig. 1).

The study was approved by the Ethics Committee of Clinico San Carlos Hospital (C.I.22/126-EC_X_Tesis) in accordance with the Helsinki Declaration and written informed consent were collected.

### Participants

Participants were recruited from three primary care centres in Spain. The population was selected via consecutive non-probabilistic sampling. The inclusion criteria were as follows: (1) confirmed diagnosis of COPD (post-bronchodilator forced expiratory volume in 1 s [FEV_1_] to forced vital capacity [FVC] ratio < 0.70) [1]; and (2) clinical stability (at least 6 weeks without exacerbations). The exclusion criteria were as follows: (1) psychiatric, cognitive or neurological disorders that make it difficult to understand orders or cooperate; (2) comorbidities that interfere with the study intervention, such as uncontrolled cardiovascular pathologies or orthopaedic disorders that negatively affect independent walking; and (3) participation in a PR programme in the last six months.

Both groups received the usual standardized pharmacological treatment for COPD at the discretion of their physician and without any intervention by the research team.

### Interventions

**Fig. 2:**
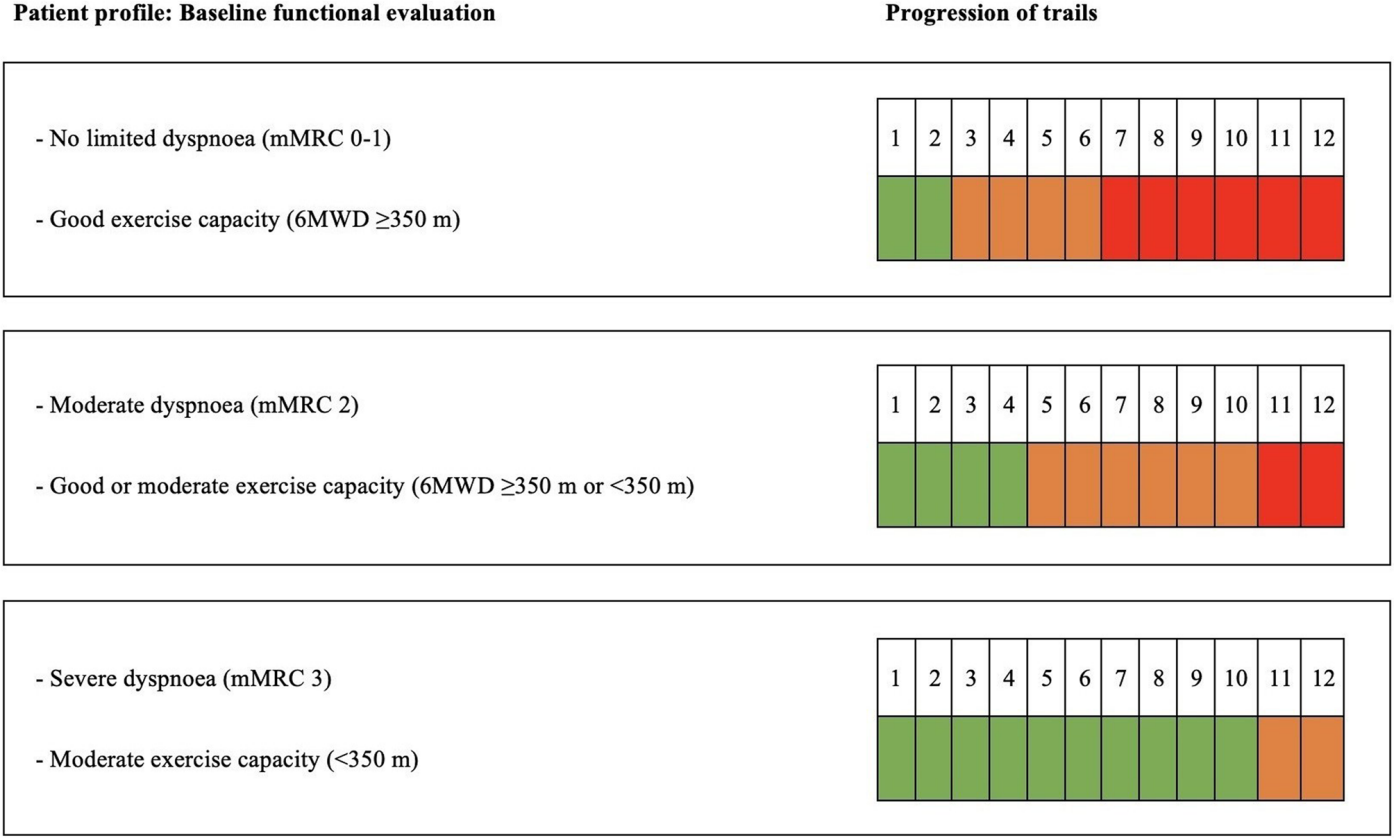
Progression in walking trail intensity during the 12-week intervention. Abbreviations: mMRC, modified Medical Research Council; 6MWD, 6-minute walk distance

The community-based multicomponent PR programme using minimal equipment was designed in accordance with the guidelines of the American Thoracic Society and the European Respiratory Society [19]. The programme lasted 13 weeks and was composed of 7 components: (1) At baseline, a structured education programme was offered during the first 4 weeks (1 time/week) where joint sessions were given to both groups and educational topics related to self-care were addressed. Accordingly, the topics addressed included the anatomy and physiology of the respiratory system, the pathophysiology of COPD, appropriate use of medication, smoking cessation, nutrition and healthy lifestyle habits, early detection of exacerbations, breathing techniques and dyspnea management, as well as social support [20–22]. The structured educational programme was conducted at the primary care center by a multidisciplinary group comprising a primary care physician, community nurse, social worker, and physiotherapist. The first educational session was given in the first week by an experienced physiotherapist who explained the principles of training, the different urban circuits, strength exercises and the main information about the intervention. During the following 12 weeks, three educational sessions were combined with exercise training sessions (3 times/week). (2) Aerobic training was conducted by walks through urban trails of different intensity represented by colour (low intensity, green; moderate intensity, orange; and high intensity, red) and designed following the Urban Training™ methodology [17]. Depending on the participant’s degree of dyspnoea and functional exercise capacity, three different progressions were established over several weeks (Fig. 2).

In all cases, participants were instructed to walk for at least 30 min along the circuit coloured according to their progression, one trail per day, ≥ 3 days per week, at a pace reaching a dyspnoea Borg scale score of 4–6 points. Depending on their functional capacity and degree of dyspnoea, participants were allowed to take rest breaks during the circuits, as all routes were equipped with benches along the way and water fountains along the way and were located near their reference healthcare centre. 3) Eight types of strength exercises were offered during the first educational session, four using elastic bands and four using body weight. Participants were informed to combine at least four of them while completing the circuit walks. Elastic bands with different resistances were provided to patients. 4) A dossier with maps trails of different intensities (low, moderate or high) available in three walkable public urban areas (boulevards and parks) was provided. 5) All participants were given an activity notebook which showed the colour of the circuit they had to complete each week; these notebooks were used by participants to record the time walked, the intensity of the training and any incidents that occurred during the training. 6) A calendar, with the participant’s individual training load progression marked on it also was provided. 7) During 12 weeks, one exercise training session per week was supervised face-to-face by the physiotherapist. The training group consisted of a maximum of 4 people who had the same degree of dyspnoea and functional exercise capacity. During the sessions, an experienced physiotherapist guided the participants through the walks and ensured that the intensity of the training was appropriate. The strength exercises were also supervised.

The TS group received all the components, except the supervised group sessions face-to-face. The TS group was telephoned by the same physiotherapist once a week. In this call, it was monitored that the participants were complying with the prescribed exercise training and positive reinforcement was given. In addition, solutions were proposed to any needs which emerged. The exercise training sessions of TS group were individual according to the guidelines provided during the first educational session. The same physiotherapist who coordinated the educational sessions was responsible for supervising both groups and was the sole provider of the exercise training sessions.

In the event that participants exhibited any warning signs, had questions regarding their medication, or required social support during the program, they were promptly offered a direct appointment with their primary care physician, nurse, or social worker, ensuring multidisciplinary support throughout the program.

### Outcome measurements

Assessments were performed at four time points: preintervention, postintervention, and six and twelve months after the end of the intervention (Fig. 1). All assessments were conducted at the healthcare centre. During visits 1, 3, 5, and 7, participants in both groups were provided with an accelerometer, which they were instructed to wear and subsequently return during visits 2, 4, 6, and 8, respectively. Data were also collected at 6 and 12 months after the end of the intervention to assess the medium- and long-term effects of the programme and to evaluate the maintenance of the outcomes achieved. This follow-up was justified by evidence indicating that, from three months onwards, the benefits of PR programmes tend to decline, approaching pre-intervention levels [23]. Data collection was obtained from all participants using standardised procedures. (1) Sociodemographic data. (2) Body mass index (3) Smoking habits. (4) Charlson Comorbidity Index [24]. (5) Lung function (forced spirometry test). (6) COPD prognosis (BODE index) [5]. (7) Degree of dyspnoea by modified Medical Research Council scale [25]. (8) Type of medication. (9) Functional exercise capacity by 6-minute walk distance (6MWD) [26]. (10) Physical activity level measured by steps/day (accelerometer Dynaport MoveMonitor, McRoberts) [27]. 11) HRQoL was collected using the COPD Assessment Test (CAT) [28]. 12) Psychological status was measured by the Hospital Anxiety and Depression Scale [29]. 13) Exercise self-efficacy was quantified by the Exercise Self-Efficacy Scale [30]. 14) Number of exacerbations was analysed based on the data recorded in the patient’s primary care medical records. 15) Adherence to treatment was evaluated by the Adherence to Treatment of Physiotherapy Scale, and attendance at the sessions was determined based on the information recorded in the participants’ activity notebook [31]. 16) Satisfaction with the study components was collected by visual analogue scale from 0 (“not satisfied at all”) to 10 points (“completely satisfied”) and 17) potential adverse events experienced during or after walks were measured [18].

### Sample size

The sample size calculation was considered as a power calculation to detect between-group differences in 6MWD (primary outcome). For this purpose, a repeated‐measured analysis of variance (ANOVA), within‐between interaction, with a power of 90% and an alpha error of 5% was chosen. Based on a previous study conducted in COPD patients with similar characteristics to the present study, a moderate small effect size (f = 0.165) was determined for improvement in 6MWD between patients who performed a supervised and unsupervised rehabilitation program [32]. Thus, 68 patients with COPD were required. Finally, a sample size of 80 participants was estimated, accounting for an expected dropout rate of 15%.

### Data analysis

Statistical analysis was performed using SPSS with a significance level of 5% (*p* < 0.05) and a 95% confidence interval. Quantitative variables are expressed as means and standard deviations, and categorical variables as frequencies. Baseline characteristics were compared using the chi-square test of independence for categorical data and the student t test for continuous data when normally distributed, or the Mann-Whitney U test for non normally distributed data. Differences between groups over time were analyzed using linear mixed models including treatment group, time, and group × time interaction. In addition, all linear mixed-effects models included baseline values of the variables to adequately adjust for initial differences. Between-group differences were assessed using ANOVA models of respective measures, and when statistically significant, multiple comparisons were performed with Bonferroni correction. At all three time points—post-intervention, 6 months, and 12 months—the 80 randomized patients were analyzed using an intention-to-treat approach. Simple imputation was used for missing values by imputing the group mean at each time point (post-intervention, 6 months, and 12 months), given the higher validity of this method [33].

## Results

**Fig. 3:**
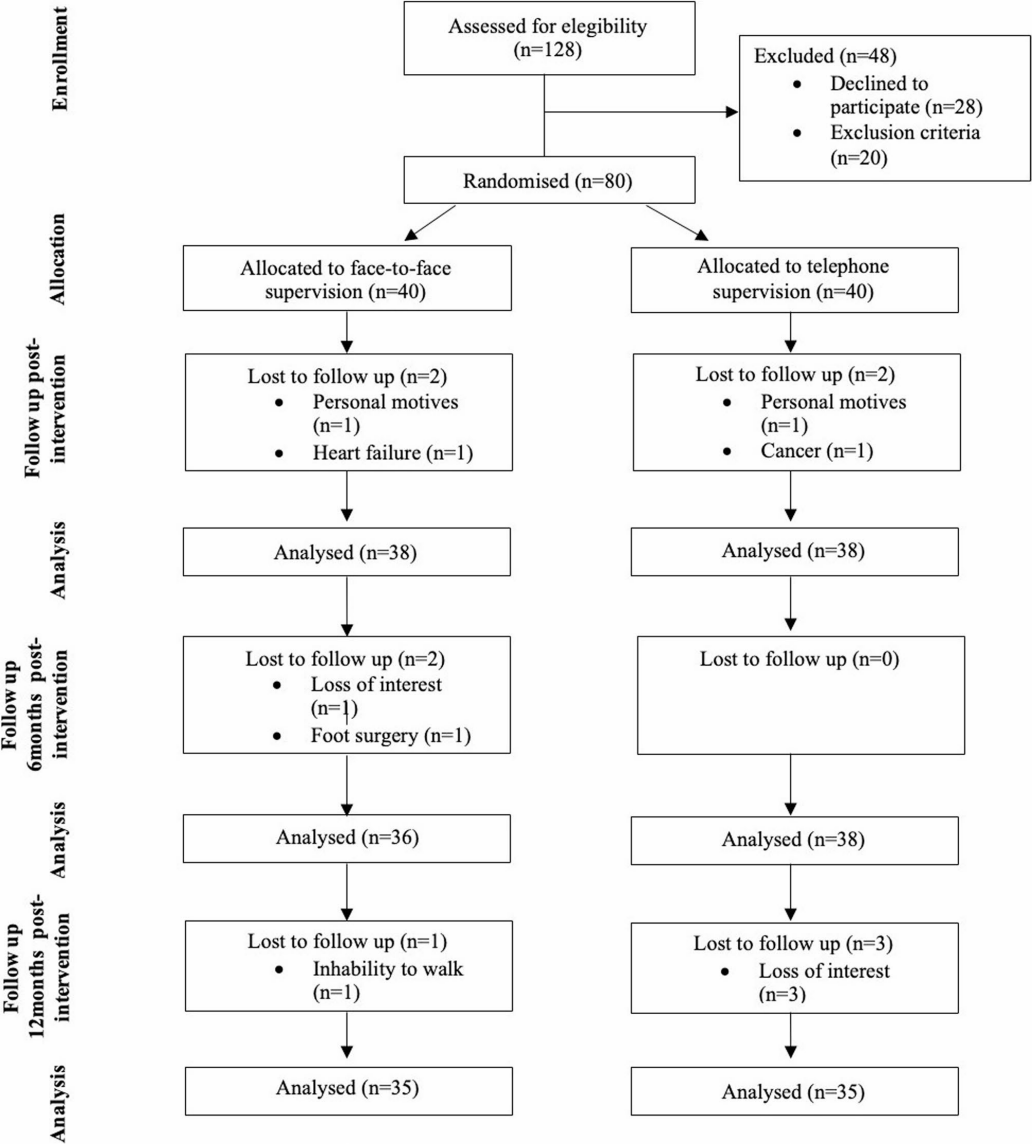
Flow chart of the participants

The study was conducted from October, 2022, to July, 2024. A total of 128 participants were evaluated for eligibility, and 80 were randomized and received the intervention. The causes of dropout and the number of participants analysed in each group are shown in (Fig. 3)

The baseline characteristics of participants are shown in Table 1. As shown in Table 1, the baseline characteristics of the two groups were generally comparable. However, statistically significant baseline differences were observed in physical activity level (steps/day) and HRQoL (CAT score), with the TS group presenting higher values in both outcomes (*p* = 0.01 and *p* = 0.006, respectively). To account for these imbalances and avoid biased estimates of treatment effects, linear mixed-effects models were adjusted by including the baseline values of these variables.

**Table 1:** Baseline characteristics

	Face-to-face supervision (*n* = 40)		Telephone supervision (*n* = 40)		*p* value
	Mean ± SD	*N*(%)	Mean ± SD	*N*(%)	
**Age (years)**	69.6 ± 8.5		68.9 ± 7.4		0.68
**Female sex**		11(27.5%)		11(27.5%)	1
**Occupation**					0.60
Active worker		7(17.5%)		7(17.5%)	
Temporary incapacity		1(2.5%)			
Retired		32(80%)		33(82.5%)	
**Body mass index** (kg/m^2^)	29 ± 5.5		28.7 ± 5.4		0.83
**Current smoker**		8(20%)		16(40%)	0.051
**Pack-year index**	49.1 ± 34.1		44.2 ± 23		0.75
**Dyspnoea** (mMRC)	1.5 ± 0.6		1.1 ± 0.6		0.059
Grade 0		1(2.5%)		5(12.5%)	
Grade 1		20(50%)		26(65%)	
Grade 2		18(45%)		9(22.5%)	
Grade 3		1(2.5%)			
**Peripheral muscle fatigue** (modified Borg scale)	3.6 ± 3.3		2.1 ± 2.6		0.052
**Comorbidities** (Charlson Index)	2.3 ± 1.4		1.9 ± 1		0.39
**Lung function**					
FEV1 (%predicted)	64.5 ± 18.9		65.7 ± 11.1		0.42
FEV1/FVC	58.4 ± 9.6		60.8 ± 6		0.51
**Airflow limitation (GOLD)**					0.26
Mild		5(12.5%)		3(7.5%)	
Moderate		28(70%)		34(85%)	
Severe		7(17.5%)		3(7.5%)	
**GOLD**					0.08
GOLD A		8(20%)		18(45%)	
GOLD B		25(62.5%)		19(47.5%)	
GOLD C		4(10%)		1(2.5%)	
GOLD D		3(7.5)		2(5%)	
**BODE index**	1.5 ± 1.3		1.1 ± 1.1		0.13
**Medications**					
Long-acting bronchodilators (LAMA/LABA)		40(100%)		39(97.5%)	0.31
Inhaled corticosteroids		25(62.5%)		21(52.5%)	0.37
**Outcomes variables**					
Functional exercise capacity: 6MWD	439.2 ± 94.6		462.5 ± 96.1		0.278
Physical activity level: Steps/day	5685.5 ± 3095.7		7740.6 ± 3811.1		0.010*
Health-related quality of life: CAT	13.7 ± 7.8		9.4 ± 5.8		0.007**
Psychological status: HADS					
Anxiety	5.1 ± 4.2		4.2 ± 3.5		0.307
Depression	3.6 ± 3		3 ± 3.5		0.413
Exercise self-efficacy: ESE	1260.5 ± 405.1		1301 ± 371.9		0.643
Exacerbations	0.6 ± 0.8		0.3 ± 0.6		0.07

*SD *Standard deviation, *mMRC *Modified Medical Research Council, *FEV1 *Forced expiratory volume in the first second, *FVC *Forced vital capacity, *GOLD *Global Initiative for Chronic Obstructive Lung Disease, *BODE *Body mass index, airflow obstruction, dyspnoea and exercise capacity, *LAMA *Long-acting muscarinic antagonist, *LABA *Long-acting B-agonist, *6MWD *6-minute walk distance, *CAT *COPD Assessment Test, *HADS *Hospital Anxiety and Depression Scale, *ESE * Exercise Self-Efficacy Scale

* *p* value < 0.05

**p value < 0.01

Some incidents were recorded during the intervention (joint pain, 6.3%; muscle pain, 13.8%). The degree of participant satisfaction with the intervention was high in both groups (FFS, 9.6 ± 0.8 points; TS, 9.5 ± 0.9 points).

Tables 2 and 3 show the differences between the groups at the end of intervention and during the follow-up period.

**Table 2:** Between-group differences in primary and secondary outcomes in face-to-face supervision and telephone supervision groups. Intention-to-treat analysis

	Face-to-face supervision *n* = 40			Telephone supervision *n* = 40			Mean difference (95% CI)		
	End of rehabilitation	6 months	1 year	End of rehabilitation	6 months	1 year	End of rehabilitation	6 months	1 year
**6MWD**	485.3 ± 94	480.7 ± 102.2	486.2 ± 108.9	479.7 ± 98.7	484.8 ± 101	481.4 ± 109	28.8 (12.3 to 45.3)**	19.15 (2.7 to 35.6)*	28 (11.5 to 44.5)**
Steps/day	7459.5 ± 3850.7	7022.4 ± 3887.2	6905.4 ± 3885.9	7861.2 ± 3500.6	8012.1 ± 3366.1	7493.1 ± 3778.4	1653.4 (377.6 to 2929.1)*	1065.3 (− 210.5 to 2341.1)	1467.3 (191.5 to 2743.1)*
CAT	8.6 ± 5.8	9.2 ± 6	7.7 ± 5.4	8.5 ± 6.1	7.9 ± 5.5	8 ± 5.2	−4.2 (− 6.3 to − 2.2)**	−3.1 (− 5.1 to − 1)**	−4.6 (− 6.7 to − 2.5)**
HADS									
Anxiety	4.7 ± 3.1	3.6 ± 2.6	3.8 ± 2.6	4.3 ± 3.3	4.1 ± 3.4	3.2 ± 2.8	−0.6 (− 1.9 to 0.6)	−1.5 (− 2.7 to − 0.2)*	−0.4 (− 1.6 to 0.9)
Depression	2.5 ± 2	2.2 ± 1.8	2.6 ± 2.5	2.6 ± 2.5	2.4 ± 2.7	2.2 ± 2.3	−0.8 (− 2 to 0.3)	−0.9 (− 2 to 0.2)	−0.3(− 1.4 to 0.9)
ESE	1496.8 ± 260.7	1438.3 ± 313.4	1442.6 ± 319.6	1457 ± 274.1	1440.8 ± 307.7	1373.6 ± 436.1	80.4 (− 83.4 to 244.2)	38.1 (− 125.7 to 201.8)	109.5 (− 54.3 to 273.3)
Exacerbations	0.2 ± 0.5	0.2 ± 0.5	0.3 ± 0.7	0.2 ± 0.5	0.1 ± 0.2	0.1 ± 0.3	−0.3 (− 0.1 to 0)	−0.2 (− 0.4 to 0.1)	−0.1(− 0.4 to 0.2)
AdT-Physio	50.4 ± 3.2	51.5 ± 3.9	50.9 ± 4.8	50.3 ± 3.8	52.9 ± 3.2	51.8 ± 5.1		−1.6 (− 3.6 to 0.4)	−1.1 (− 3.1 to 0.9)

*6MWD *6-minute walk distance, *CAT *COPD Assessment Test, *HADS *Hospital Anxiety and Depression Scale, *ESE *Exercise Self-Efficacy Scale, *AdT-Physio *Adherence to Treatment of Physiotherapy Scale

*p value < 0.05

**p value < 0.01

**Table 3:** Within-group changes in primary and secondary outcomes in face-to-face supervision and telephone supervision groups. Intention-to-treat analysis

	Within-group changes (95% CI)			Telephone supervision *n* = 40		
	Face-to-face supervision *n* = 40					
	End of rehabilitation	6 months	12months	End of rehabilitation	6 months	12 months
**6MWD**	46 (34.5 to 57.5)**	41.5 (29.1 to 53.8)**	47 (32 to 62)**	17.2 (5.7 to 28.7)**	22.3 (10 to 34.7)**	19 (4 to 33.9)*
Steps/day	1774 (839.4 to 2708.5)**	1336.9 (307 to 2366.8)*	1219.9 (179 to 2260.7)*	120.6 (− 814 to 1055.2)	271.6 (− 758.4 to 1301.5)	−247.4(− 1288.3 − 793.4)
CAT	−5.2 (− 6.7 to − 3.6)**	−4.6 (− 6.3 to − 2.8)**	−6 (− 7.6 to − 4.4)**	−0.9 (− 2.5 to 0.6)	−1.5 (− 3.3 to 0.3)	−1.4 (− 3 to 0.2)
HADS						
Anxiety	−0.5 (− 1.3 to 0.4)	−1.5 (− 2.5 to 0.5)**	−1.4 (− 2.5 to − 0.2)*	0.2 (− 0.7 to 1)	−0.1 (− 1 to 0.9)	−1 (− 2.1 to 0.2)
Depression	−1.2 (− 2 to 0.3)**	−1.5 (− 2.4 to − 0.6)**	−1.1 (− 2 to − 0.1)*	−0.4 (− 1.2 to 0.5)	−0.6 (− 1.5 to 0.3)	−0.8 (− 1.8 to 0.2)
ESE	236.3 (123.4 to 349.3)**	177.8 (54.2 to 301.4)**	182.1 (24.9 to 339.4)*	156 (43 to 268.9)**	139.8 (16.2 to 263.3)*	72.6 (− 84.6 to 229.9)
Exacerbations	−0.4 (− 0.6 to − 0.2)**	−0.4 (− 0.6 to − 0.2)**	−0.3 (− 0.5 to 0)*	−0.1 (− 0.3 to 0.1)	−0.3 (− 0.5 to 0)*	−0.2 (− 0.4 to 0)
AdT-Physio		1.1 (− 0.3 to 2.4)	0.4 (− 1.2 to 2.1)		2.7 (1.3 to 4)**	1.6 (− 0.1 to 3.2)

*6MWD * 6-minute walk distance, *CAT *COPD Assessment Test, *HADS *Hospital Anxiety and Depression Scale, *ESE *Exercise Self-Efficacy Scale, *AdT-Physio *Adherence to Treatment of Physiotherapy Scale

*p value < 0.05

**p value < 0.01

### Primary outcome

The 6MWD improved at all measurement times in both groups, with greater improvements observed in the FFS group, and the differences between the groups were significant at all time points.

### Secondary outcomes

Compared with TS group, the FFS group had an increased number of steps per day at the end of the intervention and at the 12-months follow-up (1653.4, *p* = 0.01 and 1467.3 steps/day, *p* = 0.02). Compared with those in the TS group, the CAT scores in the FFS group were lower in the short, medium and long term (*p* < 0.01). Although a significant reduction in anxiety was observed in the FFS group at the six-month follow-up (− 1.5 points on the HADS anxiety subscale, *p* = 0.02), no relevant changes were detected at the end of the intervention or at the 12-month follow-up. With respect to the level of exercise self-efficacy, no differences were found between the groups. No differences in the number of exacerbations, were found between the groups. In the FFS group, the average duration of the exacerbation period was 8.6 ± 3.7 days, 71.4% were treated in primary care, 17.9% required hospitalization and 10.7% had emergency department visits. In the TS group, the mean duration of the exacerbation period was 10.6 ± 5.7 days; 73.3% were treated in primary care and 13.3% required hospitalization. There was no difference in the level of adherence to treatment, measured with the AdT-Physio scale, between the groups. 90% of the participants (*n* = 36) in the FFS group completed the treatment, attending an average of 37 ± 4.3 sessions (92.5% total sessions). A total of 87.5% of the participants (*n* = 35) in the TS group completed the intervention, attending 36.6 ± 5.8 sessions (91.5% total sessions). Finally, no statistically significant differences in attendance in the number of sessions were detected between both groups.

## Discussion

This single-blinded randomized clinical trial is the first study to evaluate the effects of different types of supervision during community-based PR programmes using minimal equipment in individuals with COPD. FFS was superior to TS for improving functional exercise capacity and HRQoL in the short, medium and long term. Furthermore, the FFS group had increased physical activity levels at the end of the intervention and at the 12-month follow-up, and anxiety levels were reduced at six-months follow-up. However, there were no differences between the groups in terms of level of depression, exercise self-efficacy, exacerbations or adherence.

With respect to functional exercise capacity, the superiority of FFS compared with TS observed in this study is consistent with the literature, in which direct supervision by a health professional has been reported as an important aspect of the success of PR programmes [34]. In the FFS group, the change in the 6MWD was very similar to obtained in traditional centre-based PR programmes (FFS group: 46 m vs. centre-based PR programmes: 44 m) [35]. Furthermore, this improvement exceeded the minimum clinically important difference (MCID) of 30 m [36] at all measurement times, whereas the TS group achieved statistically significant but not clinically relevant improvement. The difference may be because the direct supervision of health professionals during walks through urban circuits and strength exercises ensures that the necessary training intensity to achieve physiological changes and obtain greater benefits is reached [37]. The maintenance of the results observed in both groups during the follow-up period may be explained by the simplicity and accessibility of the programme, which required only minimal equipment that patients could use independently. This feature likely promoted greater adherence and facilitated the integration of exercise into participants’ daily routines within their home and community settings, thereby supporting the long-term sustainability of the rehabilitation benefits [14, 38]. However, further studies are needed to confirm this hypothesis. Moreover, the educational sessions provided to both groups improved disease-related knowledge and promoted self-care, which may facilitate the adoption and long-term maintenance of healthy lifestyle habits [39, 40].

In terms of physical activity levels, the number of steps per day increased in the FFS group in the short and long term, with values ​​that exceeded the established MCID of 1100 steps/day [41]. This result could be because the participants who received FFS achieved higher training intensities and, therefore, greater changes in their peripheral muscles, which allowed them to be more active every day. Furthermore, in the FFS group, exercise training was carried out in a group with several people, which could promote the creation of support networks and social contact with peers, which has been shown to be an important factor in staying physically active and maintaining the results obtained in PR programmes in the long-term [42, 43].

In terms of HRQoL, the FFS group was superior to the TS group and managed to overcome the MCID by reducing the score on the CAT scale by at least four points at all measurement times compared to TS [44]. The superiority of the FFS group is consistent with the results of the study conducted by Arbillaga-Extxarri et al. [18], which concluded that telephone follow-up during exercise training was insufficient to improve HRQoL. The absence of improvement in the TS group could be expected considering that there were no changes within the group in physical activity levels or psychological state, which translates into physical inactivity and greater states of anxiety and depression, which have been associated with worse HRQoL scores in individuals with COPD [45, 46]. These results contrast with a review that suggested that telerehabilitation could be used to achieve improvements similar to those of supervised conventional PR programmes [13]. However, there is a wide range of telerehabilitation models, and the interventions using these models are heterogeneous, making comparisons difficult. Thus, interventions that use advanced telehealth technologies with internet platforms, such as websites and mobile applications, have proven to be as beneficial as centre-based PR programmes and are hardly comparable to simple TS [47].

With respect to the psychological state and exercise self-efficacy, the results agree with those of Holland’s study [48], in which there were no differences between the FFS group and the TS group in the short or long term. The lack of changes in anxiety and depression levels could be explained, in part, by the good psychological baseline status of the participants. Although a significant reduction in anxiety was observed in the FFS group at the six-month follow-up (− 1.5 points on the HADS anxiety subscale; *p* < 0.05), no relevant changes were detected at the end of the intervention or at the 12-month follow-up. However, this finding may lack clinical significance when considering that the mean anxiety and depression scores in both groups remained below 6 points at all assessment times. Given that the HADS uses a threshold of ≥ 12 points to define a case of clinical anxiety or depression, these statistically significant differences may not necessarily reflect clinically meaningful changes in the participants [49]. These findings may be further supported by the fact that the observed value did not exceed MCID [50]. Regarding exercise self-efficacy, no significant differences were observed between the face-to-face supervision (FFS) and telephone supervision (TS) groups at any of the assessment time points. This result suggests that the type of supervision did not influence participants’ confidence in their ability to engage in regular physical activity. This may be explained by the fact that self-efficacy beliefs are influenced not only by physical training, but also by a range of psychosocial factors—such as personal problems, social support, emotional state, and weather conditions—which may not have been directly modified by the intervention [22, 51]. Moreover, baseline self-efficacy scores were relatively high in both groups, which may have limited the observable impact of the intervention on this variable.This is consistent with previous studies indicating that, in patients with COPD, changes in self-efficacy levels may require more specific behavioural strategies, such as cognitive restructuring or goal-setting reinforcement, beyond structured exercise alone [52]. Therefore, future interventions may consider incorporating a psychological approach into the physical training and health education programme, aimed at specifically addressing and working on these aspects.

Regarding the number of exacerbations, no differences were found between the groups, which agrees with the findings of the systematic review by Cox et al. [13]. Considering that exacerbations are triggered by lower respiratory tract infections caused by viruses or bacteria and that the frequency of these infections is associated with an increase in systemic inflammation and physiological changes in the airways, it is reasonable to think that the type of supervision does not affect the number of exacerbations [53, 54]. Furthermore, the structured education provided to both groups regarding healthy lifestyle habits, vaccination, medication use, and the management and early detection of exacerbations may have contributed to the absence of differences between them [1, 55].

Finally, the high rate of adherence throughout the intervention in both groups could be because carrying out a PR programme in the community environment near the patient’s home avoids having the need for travel, which is one of the reasons for the lack of adherence to traditional programmes [12].

The main limitation of the study is that the majority of participants had moderate COPD, which may limit the generalizability of the results to individuals with more severe disease. This imbalance could be due to the fact that patients with advanced disease and greater disability are usually followed up in hospital settings. Additionally, this study used simple imputation for missing data instead of more robust methods such as multiple imputation. Simple imputation was chosen because of its ease of implementation, the small amount of missing data across the three time points, and to facilitate better understanding of the results. However, multiple imputation, although more complex, could have provided more accurate and less biased estimates by accounting for the variability and uncertainty inherent in missing data. Future research should consider applying this intervention to patients with more advanced COPD and explore the use of more sophisticated imputation methods to improve result validity.

FFS by a physiotherapist seems to be a key factor in improving functional exercise capacity, physical activity levels and HRQoL in individuals with COPD; both groups maintained adherence at all measurement time points. Thus, FFS during the intervention demonstrated benefits for patients, and monitoring and feedback during treatment were key strategies to maintain the results obtained at the end of the intervention. The application of this intervention was well tolerated by participants, with only minor adverse events reported, such as muscle and joint pain, suggesting it may be a safe option in a community setting. The sustained improvements observed at 6 and 12 months also support its potential as an effective long-term strategy for people with COPD. PR programmes using minimal resources address the main treatable features of COPD by promoting self-management of the disease and could be a particularly useful treatment strategy in populations with limited access to hospital services.

The results of this study highlight the potential of community-based PR using minimal equipment as a reproducible and cost-effective strategy, particularly in settings with limited healthcare infrastructure or economic resources. Implementing interventions based on urban walking circuits, bodyweight and elastic band exercises, and group-based supervision within patients’ local environments reduces reliance on hospital facilities and specialized equipment. This approach may also be beneficial for patients who face difficulties traveling to hospital-based programs due to work commitments, caregiving responsibilities, or personal limitations. It may be especially valuable in rural areas or low- and middle-income countries, where access to conventional PR programs is often limited. In future lines of research, it would be valuable to investigate the cost-effectiveness of this intervention across diverse healthcare settings, utilizing a larger and more heterogeneous patient population to enhance the generalizability of the findings.

## Conclusions

FFS during a community-based PR programme using minimal equipment was superior to TS in improving functional exercise capacity, physical activity levels and HRQoL in the short and long term in individuals with COPD. However, no changes were detected in terms of depression status, exercise self-efficacy, number of exacerbations or adherence. The results of this study support the safety of community-based PR programmes and the importance of adding FFS to obtain greater benefits.

## Supplementary Information

Supplementary Material 1

## Data Availability

The datasets used and/or analysed during the current study are available from the corresponding author on reasonable request.

## Abbreviations

COPD: chronic obstructive pulmonary disease
HRQoL: health-related quality of life
PR: Pulmonary rehabilitation
TS: telephone supervision
FFS: face-to-face supervision
6MWD: 6-minute walk distance
CAT: COPD Assessment Test
MCID: minimum clinically important difference
